# From Prediction to Prevention: The Intricacies of Islet Autoantibodies in Type 1 Diabetes

**DOI:** 10.1007/s11892-025-01595-1

**Published:** 2025-06-24

**Authors:** Aye Khine, Zoe Quandt

**Affiliations:** 1 Division of Diabetes, Endocrinology and Metabolism, Department of Medicine, University of California San Francisco, San Francisco, CA 94143 USA; 2 Diabetes Center, University of California, San Francisco, San Francisco, CA 94143 USA

**Keywords:** Type 1 diabetes, Autoantibodies, Screening

## Abstract

**Purpose of Review:**

This review synthesizes current knowledge on islet autoantibodies (IAs) as predictive biomarkers for type 1 diabetes (T1D), focusing on their role in disease staging, autoantibody patterns, advancements in screening methodologies, and the implications of implementing population-wide screening initiatives.

**Recent Findings:**

Autoantibody profiling has refined T1D risk stratification, with progression rates influenced by IA characteristics including number, type, order of appearance, and affinity. While screening efforts initially targeted genetically at-risk groups, approximately 90% of new TID diagnoses occur in individuals without a family history, underscoring the need for broader population-based screening. The approval of teplizumab, a therapy shown to delay clinical T1D onset, represents a paradigm shift by providing an intervention following early identification through screening. Technological advancements have further optimized IA detection and therapeutic strategies. However, challenges such as cost-effectiveness, implementation logistics, and assay standardization remain.

**Summary:**

T1D is a chronic autoimmune disorder characterized by progressive pancreatic beta-cell destruction, leading to insulin deficiency. The natural history of T1D is typically marked by the appearance of IAs long before clinical symptoms emerge, providing a window for early detection and intervention. Identifying at-risk individuals during this asymptomatic phase can reduce disease severity at clinical onset and facilitate timely application of disease-modifying therapies like teplizumab. Emerging evidence emphasizes that IA characteristics collectively shape disease risk and progression. Advancements in screening technologies and therapies continue to support the integration of IA screening into clinical care, marking a significant step toward effective T1D prevention and management.

## Introduction

Type 1 Diabetes (T1D) is an autoimmune disorder characterized by the progressive destruction of pancreatic beta cells, leading to insulin deficiency [1]. In 2021, 8.4 million individuals worldwide were estimated to have T1D [2]. While T1D was traditionally considered a childhood diagnosis, most new cases actually occur in adults. One study revealed that among all T1D patients, 57% were diagnosed after the age of 40, and 83% were diagnosed at age 20 years or older [3]. The global prevalence of T1D is projected to rise significantly, with estimates predicting a 60–107% increase by 2040 [2]. Despite data suggesting that the diagnosis of T1D is more common in adults than in children, research on the adult population remains comparatively limited.

**Fig. 1:**
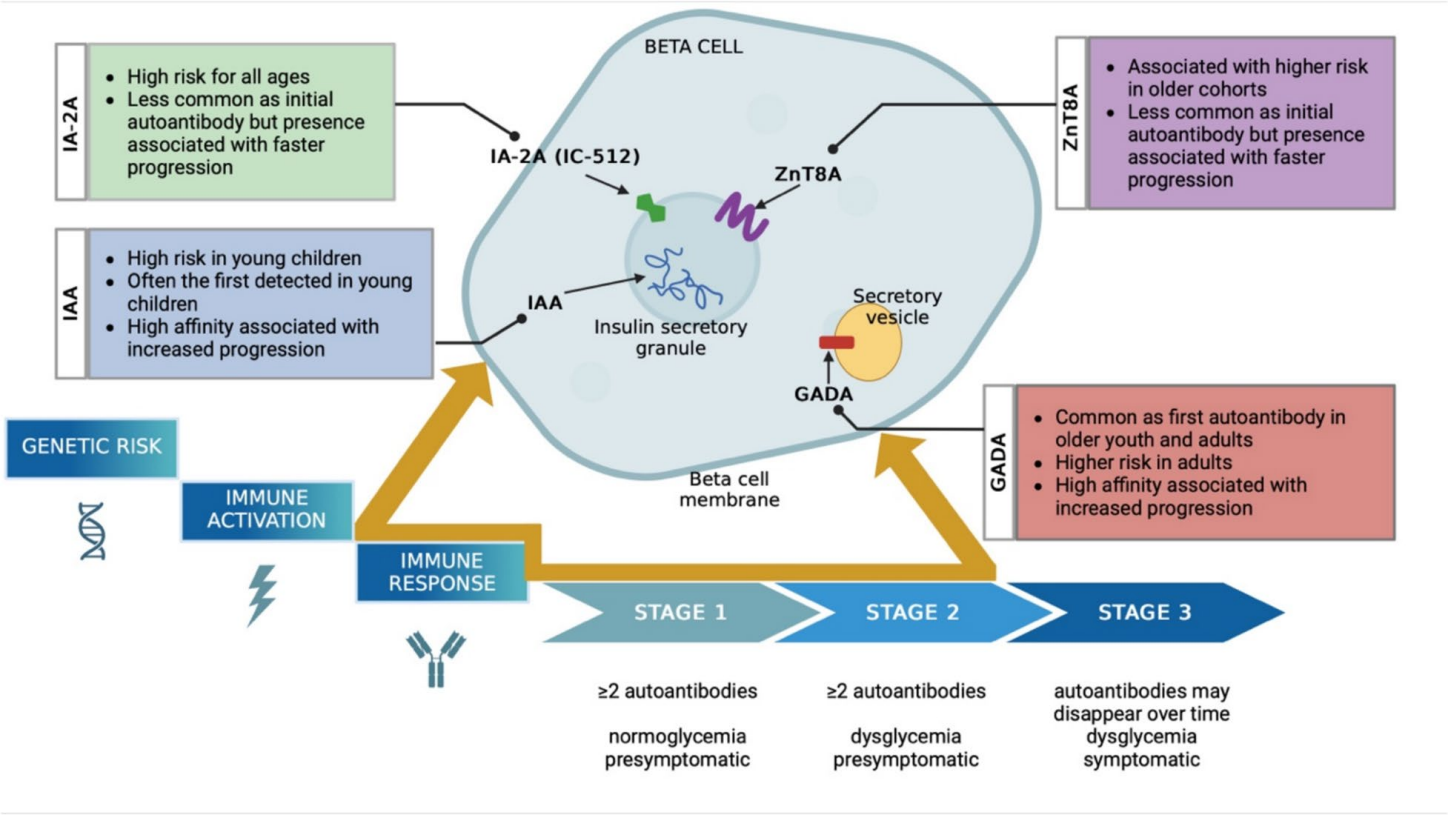
Schematic of T1D progression from genetic susceptibility to clinical diagnosis, overlaid with the features of pancreatic beta-cell destruction and islet autoantibody (IA) appearance. It highlights key IAs implicated in T1D, including insulin autoantibody (IAA), glutamic acid decarboxylase antibody (GADA), islet-antigen-2 antibody (IA-2A, IC-512), and zinc transporter 8 antibody (ZnT8A). The beta-cell graphic visually represents the autoimmune changes that are thought to occur during the immune response phase and early stages of T1D. Abbreviations: IAA (insulin autoantibody), GADA (glutamic acid decarboxylase antibody), IA-2A/IC-512 (islet-antigen-2 antibody), ZnT8A (zinc transporter 8 antibody). Modified from The Stages of Type 1 Diabetes by Type1Strong (https://www.type1strong.org/blog-post/the-stages-of-type-1-diabetes) and created in https://BioRender.com

The natural history of T1D is typically marked by the appearance of islet autoantibodies (IA) long before clinical symptoms arise. This preclinical phase offers a critical window for early detection and intervention, especially with the approval of disease-modifying therapies that can delay or even prevent progression to clinical T1D [4]. Longitudinal cohort studies have advanced our understanding of autoantibody screening and strategies to implement generalized population screening programs (Fig. 1). These studies have mainly targeted high-risk groups, such as relatives of those with T1D. However, ~ 90% of people diagnosed with T1D do not have a family history of T1D, underscoring the need for the expansion of screening to identify more individuals in the early stages of disease [5].

Advances in understanding the natural history of autoantibodies, combined with innovations in screening methods, have expanded opportunities for effective screening programs. This review examines the role of autoantibodies in diagnosing T1D, with a focus on their utility in staging T1D, the effectiveness of screening methods, and implications of generalized population screening. It also explores the cost-effectiveness of screening strategies and future directions to enhance early diagnosis and management.

## Diagnostic Criteria and Staging for T1D

Studies of the natural history of T1D and discovery of islet autoantibodies have led to a paradigm shift in the approach to T1D, most notably the use of progression through defined stages [6]. Stage 1 T1D is defined as the presence of two or more autoantibodies with normal glucose levels and no symptoms. At stage 1, the risk of progressing to clinical (stage 3) T1D within 5 years is 43.5%, 10 years is 69.7%, and 15 years is 84.2% when reviewed in a combined analysis [7]. Stage 2 T1D involves two or more autoantibodies and dysglycemia without symptoms. Per American Diabetes Association (ADA) guidelines (2024), dysglycemia is defined by impaired fasting plasma glucose 100–125 mg/dL, 2-h oral glucose tolerance test (OGTT) plasma glucose 140–199 mg/dL, or hemoglobin A1C (HbA1c) 5.7–6.4% [8]. In this stage, the risk of progression to clinical T1D is 60% within 2 years. Within 5 years, the risk of progression is 75% with a positive predictive value of 96% [9]. Stage 3 T1D, marked by persistent hyperglycemia and the onset of symptoms (such as polyuria, polydipsia, weight loss, fatigue, or diabetic ketoacidosis) may involve one or more autoantibodies, as autoantibodies can become undetectable over time [6]. Early identification of high-risk individuals, based on autoantibody presence and glucose tolerance, provides an opportunity for timely intervention.

## Natural History of Autoantibodies in T1D

Multiple longitudinal studies have shown that the appearance of islet autoantibodies precedes clinical (stage 3) T1D, however the rate of progression from seroconversion to clinical disease remains highly heterogeneous [10]. Known islet autoantibodies include insulin autoantibody (IAA), glutamic acid decarboxylase antibody (GADA), islet-antigen-2 antibody (IA-2A also known as IC-512A) and zinc transporter 8 antibody (ZnT8A). Important information about islet autoimmunity has been obtained from genetically at-risk cohorts followed over time. Studies recruiting from birth include the Diabetes Autoimmunity Study in the Young (DAISY), the Finnish Type 1 Diabetes Prediction and Prevention (DIPP) study, the BABYDIAB study, and The Environmental Determinants of Diabetes in the Young (TEDDY) study. Data from the Diabetes Prevention Trial-Type 1 (DPT-1) and the Type 1 Diabetes TrialNet Pathway to Prevention (PTP) Study also have provided further data by screening first and second-degree family members (age 1–45 years) of probands with T1D. Key features of these cohorts are highlighted in Table 1. All of these studies consistently showed that individuals with multiple autoantibodies have a high risk of progressing to clinical T1D. Additionally, age at seroconversion and specific autoantibodies present can influence the speed of disease progression, making early detection pivotal in identifying those most at risk [10, 11]. Accurate prediction of the most rapidly progressing individuals is important for risk assessment, and ultimately for identification of those more appropriate for disease modifying therapy.

**Table 1:** Selected cohort studies of individuals genetically at risk for T1D. This table is representative and not exhaustive of all cohort studies

Study	Recruitment Duration	Location(s)	Goals	Population
BABYDIAB	1989–2000	Germany	To understand the natural history of autoantibodies, genetic and environmental factors, examine what IA characteristics were most associated with progression to T1D	1,650 children of parents with T1D. Followed with repeat autoantibody screening
Diabetes Autoimmunity Study in the Young (DAISY)	1993–2004	US (Colorado)	To understand the natural history of T1D autoimmunity and the genetic/environmental factors	31,881 newborns screened for high-risk HLA genotypes. Follows 2,547 high-risk children and first-degree relatives from birth until first documentation of T1D or up to age 30 years
Diabetes Prevention Trial-Type 1 (DPT-1)	1994–2003	US, Canada	To test whether insulin administration could prevent or delay the onset of T1D	First and second-degree relatives of individuals with T1D at increased risk based on IA status and HLA genotype. 103,000 relatives screened and 3,500 enrolled for follow-up
Diabetes Prediction and Prevention Project (DIPP)	1994-ongoing	Finland	To understand the natural history of IA, assess environmental risk factors, and test preventive strategies for T1D	Newborns with high-risk HLA genotypes identified through population-based genetic screening (cord blood). Screened > 250,000 and high-risk enrolled for follow-up
Diabetes Prediction in Skane (DiPiS)	2000–2004	Sweden	To investigate prevalence of genetic risk of T1D, natural history and risk factors	Newborns with high-risk HLA genotypes identified through population-based genetic screening
The Environmental Determinants of Diabetes in the Young (TEDDY)	2003–2010	US (Colorado, Georgia, Florida, Washington) and Europe (Germany, Sweden, Finland)	Identify environmental factors that trigger or protect against the development of IAs or T1D in genetically susceptible individuals	424,000 newborns < 4 months of age screened for high-risk HLA genotypes or first-degree relative affected with T1D. Follows 8,676 at-risk newborns (89% general population, 11% first-degree relatives)
TrialNet Pathway to Prevention (PTP)	2004-ongoing	US, Canada, Finland, UK, Italy, Germany, Sweden, Denmark, Australia, New Zealand	To identify at-risk individuals and test interventions to delay or prevent the onset of T1D	Ages 1–45 years who have an immediate family member (child, parent, sibling) with T1D. Ages 1–20 years who have an extended family member with T1D. > 200,000 relatives screened

### Age of Autoantibody Appearance

Autoantibody seroconversion in genetically at-risk children typically occurs at a young age, with prominent peaks in early childhood. The TEDDY study found that the seroconversion rate was highest at age 6 months to 3 years, peaking at 9 months. This peak was notably evident for IAA as the initial autoantibody with progression to multiple autoantibodies [12]. The BABYDIAB study found that the highest incidence of autoantibody seroconversion in genetically at-risk children occurs between 9 months and 2 years of age [13]. Similarly, the DIPP study reported that children with HLA-conferred susceptibility to T1D who progressed to clinical T1D before puberty were most likely to seroconvert before age 4 years, with a peak seroconversion rate at age 2 years [14]. This early onset of seroconversion has been shown to correlate with faster progression to T1D, particularly in children who develop multiple islet autoantibodies before the age of 3 years [7]. 

Median ages for specific autoantibody appearances support the association of earlier seroconversion with a higher risk of progression to clinical T1D. IAA tends to appear within the first two years of life, while GADA appears between ages 3 and 5 years in children with increased genetic risk or a family history of T1D. IA-2A and ZnT8A generally appear after the initial autoantibody seroconversion. The TEDDY study found that the median age at initial IAA-only seroconversion was 1.83 years, while GADA-only seroconversion occurred later, with a median age of 4.28 years. When GADA emerged as a second autoantibody, it appeared around 3.7 months after the first autoantibody; in contrast, IAA as a second autoantibody had a median onset of 5.9 months. The development of IA-2A or ZnT8A as a second autoantibody had a median onset of 13.3 months. The overall median time between the appearance of the first and second autoantibodies was 6.8 months, with no significant differences based on whether GADA or IAA was the first to appear [12].

Risk of T1D progression strongly relates to age. TrialNet found that the most important factor associated with a more rapid rate of progression is age. Children with multiple autoantibodies progressed to clinical T1D more rapidly than adults [15]. 

### Number of Autoantibody Appearance

Increased number of autoantibodies correlates with higher risk and faster progression to T1D. Pooled data from DAISY, DIPP, and BABYDIAB showed that in genetically at-risk children, progression to T1D within 10 years in those with no autoantibodies was 0.4%, one autoantibody was 14.5%, and multiple autoantibodies was 69.7%. Progression to clinical T1D after multiple autoantibody seroconversion was 43.5%, 69.7%, and 84.2% at 5, 10, and 15 years of follow-up. The lifetime risk of clinical T1D approaches 100% once two or more islet autoantibodies are detected [7]. 

In contrast, the TEDDY study reported on the 5-year risk of clinical T1D. Within this population, the 5-year risk was 11% with one autoantibody, 36% with two autoantibodies, and 47% with three autoantibodies [16]. In relatives of individuals with T1D, the DPT-1 study found that the 5-year risk of developing clinical T1D was 25% for two autoantibodies, 40% for three autoantibodies, and 50% for four autoantibodies [17]. Of note, these rates were noted to be lower than in the larger pooled cohort described above. The Fr1da study found significantly greater risk associated with children who had 4 autoantibodies rather than 2 autoantibodies (Hazard Ratio (HR) 1.85) [18].

The TrialNet study revealed that more than 85% of autoantibody-positive relatives with impaired glucose tolerance develop clinical T1D within 5 years [15]. A multicenter retrospective study of 3,015 first-degree relatives found that progression risk is strongly influenced by age and the number of autoantibodies. Younger relatives (age < 20 years) with multiple autoantibodies had the highest progression risk, with a 52.9% risk of developing T1D within 5 years and 82.3% risk within 10 years. In contrast, individuals with only one autoantibody or older age (age ≥ 20) exhibited lower progression rates. The 20-year diabetes risk was 91.2% if both high-risk factors (age < 20 and multiple autoantibodies) were present but decreased to 59.9% with one risk factor and 35.7% for relatives age ≥ 20 with only one autoantibody [19].

In addition, studies have shown that once multiple-autoantibody positivity has developed, individuals from the general population have comparable risk of T1D development as those with a family history of T1D, therefore insights from prior genetically selected cohorts may hold relevance to the general population [18].

### Type and Order Autoantibody Appearance

The sequence and type of autoantibody appearance play a critical role in determining the risk and rate of progression to clinical T1D. IAA is often the first detected autoantibody in young children, with its incidence declining with age. The DAISY study reported that children with persistently positive IAA progressed to T1D more rapidly, with 100% developing T1D by 5.6 years, compared to 63% in those with fluctuating IAA levels over a 10-year follow-up period. Age at first autoantibody appearance and IAA levels were major predictors of T1D diagnosis, while GADA and IA-2A did not have the same predictive strength in this cohort [20]. Children with IAA as the first autoantibody were found to have a higher risk of progression (10-year risk of 71.4%, 15-year risk of 82.7%, 20-year risk of 92.1%) compared to those who are without IAA in the first positive sample (10-year risk of 47.6%, 15-year risk of 53.9%, 20-year risk of 61.6%) [11].

In contrast, GADA is typically the first autoantibody in older youth and adults. In younger individuals, GADA is associated with slower disease progression, whereas in older cohorts, its presence correlates with significantly higher risk of clinical T1D. TrialNet’s PTP study found that single GADA positivity was associated with an increased risk of progression to multiple autoantibodies and clinical T1D, with adults aged 45 years exhibiting a fourfold greater risk of progression than young children. This highlights the age-dependent impact of GADA, where its prevalence and influence on disease progression are more pronounced in adults [21].

The differences in IA in adults extend beyond this finding. While IAA often initiates the cascade towards multiple autoantibody positivity in children, GADA more frequently appears alone and remains stable if it does not progress further. IA-2A and ZnT8A are less common as the initial autoantibodies, but their presence is strongly associated with faster progression to T1D compared to when both autoantibodies are absent. In first-degree relatives of individuals with T1D, IA-2A and/or ZnT8A is associated with a 5-year progression rate to clinical T1D of 45% [22]. IA-2A as a second autoantibody was associated with a significantly greater risk of progression compared to IAA, GADA, or ZnT8A, independent of initial autoantibody type [12].

Several studies have examined the relationship between GADA titers and clinical features of T1D. Higher GADA titers have been associated with lower urine C-peptide levels, greater insulin dependence, and higher frequency of autoimmune thyroid disease [23, 24]. Elevated GADA titers at the time of T1D diagnosis have also been linked to an increased risk of long-term complications such as diabetic retinopathy [25]. In a study of GADA-positive individuals with type 2 diabetes (T2D), those with high GADA titers (> 32 arbitrary units) demonstrated more pronounced features of insulin deficiency, including higher HbA1c, lower BMI, lower total cholesterol, and a lower prevalence of metabolic syndrome compared to those with low GADA titers (≤ 32 arbitrary units). These findings suggest that high GADA titers may reflect a more severe autoimmune phenotype within increased beta cell destruction. Additionally, higher GADA levels were associated with increased IA-2A positivity, further supporting the link between titer levels and autoimmune burden [23]. Recent efforts, such as the Type 1 Diabetes Intelligence (T1DI) study, have advanced the understanding of autoantibodies by attempting to create phenotypic clusters based on their association with T1D risk [11]. In this study, IAA was typically the first autoantibody to appear (median age 1.6 years), followed by GADA (1.9 years), and IA-2A (2.1 years). The highest risk of T1D was observed in children positive for all three autoantibodies, with a 5-year risk of 69.9% and 10-year risk of 89.9%. Children with persistent IAA and GADA had the second-highest risk, with a 5-year risk of 39.1% and a 10-year risk of 73.8%. Notably, this finding suggests that while IA-2A is a strong predictor of T1D in young children, its presence is not essential for disease progression. Clusters characterized by persistent GADA and IA-2A had a 5-year risk of 30.9% and 10-year risk of 68.2%. In this group, IAA often appeared early but reverted during follow-up, with clinical T1D developing later (around age 9 years). However, findings regarding IAA reversion have varied [26]. Late persistent GADA positivity showed lower risk, with a 5-year risk of 10.5% and a 10-year risk of 24.7%. A single, often reverting, antibody was associated with significantly lower risks (5-year risk of 1.6% and 10-year risk of 4%).

### Assay Types for Autoantibody Detection

Screening for islet autoantibodies has evolved considerably, with various assay techniques now in use. Clinical trials and research protocols often confirm autoantibody positivity with repeat testing to minimize false positives, which is particularly critical in populations with low disease incidence or when considering interventions. Traditional methods, such as radioimmunoassay (RIA), have been integral to autoantibody detection and are considered the “gold standard” [27]. This approach, often used in research settings, involves radiolabeled antigens to detect antibody-antigen complexes. Newer assay technologies include electrochemiluminescence (ECL), enzyme-linked immunosorbent (ELISA), luciferase immunoprecipitation (LIPS), and antibody detection by agglutination PCR (ADAP) [28].

ECL assays are increasingly recognized for their high sensitivity and specificity, and they have also demonstrated improved prediction for T1D progression over RIA. In TrialNet, individuals with autoantibodies detected by RIA underwent further analysis with ECL. Those positive for GADA and/or IAA by ECL had a 34–58% risk of progressing to clinical T1D within six years, compared to a 5% risk for ECL-negative individuals. Most ECL-negative cases were single-autoantibody positive by RIA, underscoring ECL’s enhanced discrimination capacity [29]. ECL assays have also been adapted into multiplex platforms which can detect multiple IAs (IAA, GADA, IA-2A) and tissue transglutaminase antibody (TGA) simultaneously [30]. Other approaches, such as ELISA, offer accessibility and cost-effectiveness, as seen in the Fr1da study, which used a 3-screen ELISA for initial GADA, IA-2A, and ZnT8A detection [18]. Emerging assays like ADAP and LIPS provide novel methodologies. ADAP amplifies DNA-antigen conjugates for highly sensitive detection, while LIPS uses luminescence to quantify serum antibodies. ADAP has been adapted to a 5-plex assay that combines all 4 islet autoantibodies (IAA, GADA, IA-2A, ZnT8A) and TGA [31]. It is being used by the Antibody Detection Israel Research (ADIR) general population screening program and was found to be comparable in performance to standard RIA and ELISA [32]. In addition, LIPS assays for IAA, GAD, IA-2A, and ZnT8A were found to be comparable to RIA [33–35]. Within an autoantigen, there are also epitopes that are disease-relevant and improve disease prediction in at-risk individuals. Details on antibody epitopes and further differences in assays has previously been reviewed [36].

Further studies are needed to directly compare these different assays, especially regarding their advantages and disadvantages as we progress towards general population screening. The first international workshop to attempt standardization of insulin autoantibodies was in 1987 [37]. Additional evaluations were done by the Diabetes Autoantibody Standardization Program (DASP) [38], which was then superseded by the Islet Autoantibody Standardization Program (IASP). The IASP works to assess assay proficiency, harmonize interlaboratory measures, evaluate novel assays, and highlights the ongoing heterogeneity and lab-specific dependence of results [27].

### Affinity of Autoantibodies

Affinity refers to the strength of binding of an antibody to its binding site. Predictive value of autoantibodies increases when measured by high-affinity methods. High affinity of IAA has been associated with progression to multiple autoantibodies and clinical T1D. In the BABYDIAB cohort, high affinity of IAA was associated with HLA DRB1*04, younger age of IAA appearance, and subsequent progression to multiple islet autoantibodies or T1D. IAA affinity in multiple antibody-positive children was on average 100-fold higher than in children who remained single IAA positive or became autoantibody negative [39]. Another study from Germany found that using a threshold of ≥ 10^9^ l/mol, 22 of the 24 children who developed multiple islet autoantibodies or diabetes were correctly identified by high-affinity IAA and 18 of 22 who did not develop multiple islet autoantibodies or diabetes were correctly identified by low-affinity IAA [40].

Similar to IAA, high-affinity GADA was also associated with multiple autoantibodies and the development of clinical T1D. GADA affinity was higher in multiple autoantibody-positive children and in HLA DR3-positive children [41, 42]. Additional studies are needed on autoantibody affinity to further characterize these findings. Interestingly, despite IA-2A’s importance in predicting clinical T1D in other studies, one study of IA-2A affinity was not associated with progression to clinical T1D or HLA haplotype [43].

## Screening Approaches

**Fig. 2:**
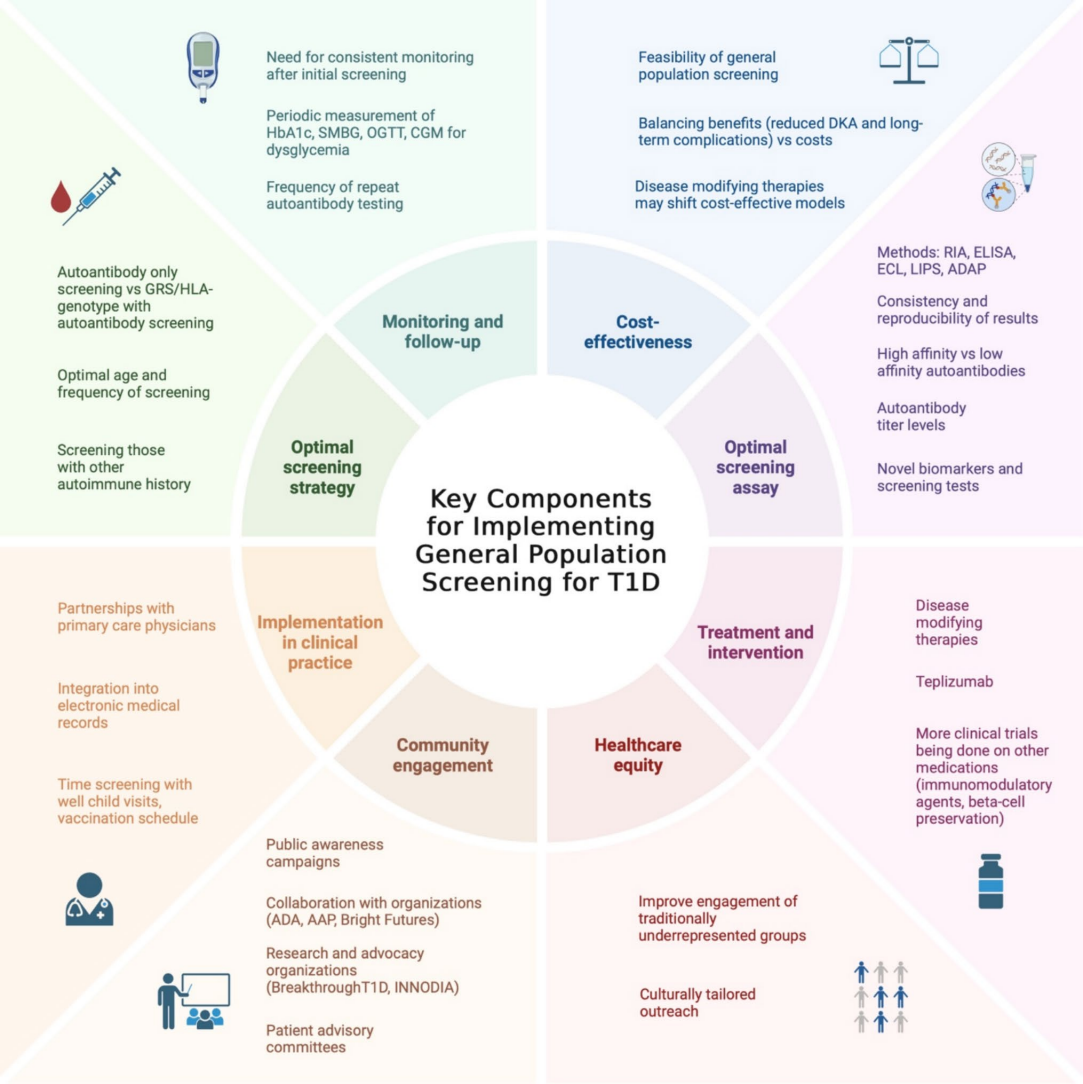
Key components and considerations for implementation of T1D general population screening. Created in https://BioRender.com

Generalized population screening for T1D has evolved significantly, recognizing that ~ 90% of newly diagnosed individuals do not have a family history of T1D [5]. Initial studies focused on genetically at-risk individuals, defined by having a first-degree relative with T1D or high-risk HLA genotypes which is statistically beneficial as it increases the disease incidence. The World Health Organization’s criteria for generalized screening, such as the importance of detecting early-stage disease and the availability of diagnostic and therapeutic options, have been further met with the approval of teplizumab as an early-stage T1D intervention but decisions on how to implement screening are ongoing given the complexity (Fig. 2) [4, 44].

Some screening programs combine HLA genotyping or genetic risk scores (GRS) with autoantibody testing to identify individuals at the highest risk, which can improve cost-effectiveness. However, this approach may miss individuals with autoantibody positivity but without high-risk genotypes. International programs, such as DIPP, BABYSCREEN, and GPPAD, and US-based initiatives, including PLEDGE and CASCADE, employ combined genetic and autoantibody screening strategies [45–49].

In contrast, autoantibody-screening alone may capture more individuals but is resource-intensive and lacks clarity on optimal screening ages. In Europe, programs like EDENT1FI and Fr1da exemplify international efforts to expand generalized autoantibody screening [50, 51]. In the US, the ASK program screens children for presymptomatic T1D and celiac disease [52]. Similarly, Australia’s T1D National Screening Pilot evaluates different methods of screening, including genetic testing at birth or infancy and autoantibody testing between ages 2 and 6 [53]. Adult-onset T1D, which accounts for over half of new diagnoses, remains underexplored. Programs like T1DRA in the UK aim to address this gap by studying autoantibody prevalence and disease progression in adults aged 18–70 [54]. All of these screening programs have slight variations and approaches to population-based screening, and further details are discussed in Table 2 [55–60].

**Table 2:** Ongoing Screening Programs. This table is representative and not exhaustive of all programs

A. Selected screening programs for relatives of individuals with T1D				
Screening Programs for First Degree Relatives	Location(s)	Population Screened	Assay	Comments
DiaUnion	Denmark, Sweden	Relatives and general population	Screening: ADAP	DiaUnion 2.0 plans to establish a public screening program
INNODIA	Europe	Relatives and general population	Screening: RIA	European network dedicated to preventing and curing T1D
TrialNet Pathway to Prevention (PTP)	US, Canada, Europe, Australia	2.5 to 45 years with parent, sibling, or child with T1D 2.5 to 20 years with aunt/uncle, cousin, grandparent, nice/nephew, or half-sibling with T1D	Screening: RIA (IAA and GADA, followed by IA-2A, ZnT8A, and ICA if positive)	Objective is to identify at-risk individuals eligible for clinical trials
Type1Screen	Australia, New Zealand	Relatives age 2–30 years or previously had positive IA test	Screening: IAA: RBA or ADAP GADA, IA-2A, ZnT8A: ELISA or ADAP	IA testing for children and young adults
B. Selected generalized population screening programs with genetic prescreening with autoantibody follow-up				
Program	Location(s)	Population Screened	Assay	Comments
Australian T1D National Screening Pilot	Australia	Cohort 1: newborns Cohort 2: infants Cohort 3: age 2, 6, 10-year olds	Cohort 1: heelprick for GRS Cohort 2: saliva swab for GRS Cohort 3: dried bloodspot ADAP (IAA, GADA, IA-2A, ZnT8A)	Identifying best way to screen by testing three cohorts (newborns and infants with high genetic risk before IA testing), another cohort with IA testing only
BABYSCREEN	Finland	Newborns to 3 years with high-risk HLA genotype for T1D or celiac disease	HLA genotype followed by RIA (IAA, GADA, IA-2A, ZnT8A, TGA)	Newborns with parental consent receive cord blood HLA screening. High-risk genotypes followed by autoantibody screening for T1D and celiac disease
DIPP	Finland	Age 0.25–1.5 years with high-risk HLA genotypes	HLA genotype followed by RIA (IAA, GADA, IA-2A, ZnT8A)	Newborns with parental consent receive cord blood HLA screening. High-risk genotypes followed by IA screening
Early Check	North Carolina	Newborns < 1 month of age	GRS, follow-up IA testing depending on risk	Screens for multiple genetic conditions, including T1D
GPPAD	Germany, UK, Belgium, Sweden, Poland	Infants < 1 month of age	47-SNP GRS to identify those with > 10% risk of ≥ 2 IA by age 6 years	At-risk infants can participate in a primary prevention trial
Population Level Estimate of type 1 Diabetes risk Genes in Children (PLEDGE)	Sanford Health in US (North Dakota, South Dakota, Minnesota)	Age < 6 years or 9–16 years	Initial GRS screen, followed by IA testing Screening: RIA Confirmation: ECL	GRS with newborn screen or study entry. IA testing if at-risk, followed for T1D and celiac disease
CASCADE	US (Washington)	Newborn to 8 months or age 4–8 years	Initial GRS screen followed by IA testing (RIA, LIPS)	Initial GRS screen, at-risk followed for T1D and celiac disease
C. Selected generalized population screening programs with autoantibody screening				
Program	Location(s)	Population Screened	Assay	Comments
Autoimmunity Screening for Kids (ASK)	Colorado, expanded to US	Age 1 to < 18 years	Screening: RIA Confirmation: RIA, ECL (IAA, GADA, IA-2A, ZnT8A, TGA)	Screening for T1D and celiac disease
Fr1da	Germany	Age 1.75–10.99 years	Screening: Capillary blood multiplex three-screening ELISA (GADA, IA-2A, ZnT8A) and LIPS (IAA) Confirmation: RIA (IAA, GADA, IA2A, ZnT8A)	General population screening of children, follow up for dysglycemia depending on stage
DiaUnion	Denmark, Sweden	Steno Diabetes Center (Denmark) screens family members to T1D patients age < 40 years Lund University (Sweden) screens general population age 2–14 years	Screening: ADAP multiplex Confirmation: RIA	Started in 2020, screening for T1D, celiac disease, and autoimmune thyroid disease
Detection of T1D and Celiac Disease in the Pediatric Population (D1Ce)	Italy	Ages 2, 6, 10 years	Screening: various methods (IAA, IA-2A, GADA, ZnT8A)	Network of family pediatricians will screen 5000 children in 4 regions of Italy
Antibody Detection Israeli Research (ADIR)	Israel	Age 9–18 months and 5 years	Screening: ADAP (IAA, GADA, IA-2A)	General population screening in Israel to identify children at risk for T1D
ELSA	UK	Age 3–13 years	Screening: RSR 3-screen ELISA (IA-2A, GADA, ZnT8A) from dried blood spot	Fingerstick test followed by venous blood test for IA
T1DRA	UK	18–70 years	Screening: Dried blood spot (RSR) Confirmation: Venous blood sample (IAA, IA-2A, GADA, ZnT8A)	Followed for IA positivity, referred to clinical trials through INNODIA
EDENT1FI	Throughout Europe, with partners also in US	Age 1–17 years	Screening: 3-screen ELISA RSR	Global collaboration between 27 partners in 13 countries with common goal to arrest T1D at the pre-clinical phase

## Benefits and Challenges of Screening

There are well established benefits for screening for T1D but these are met by many challenges both in cost and logistical decisions (Fig. 2). Some of the benefits of monitoring for early stages of T1D include decreased rates of DKA at clinical T1D onset, reduced disease severity at clinical onset, early education, and opportunities for disease modifying treatments during the presymptomatic stages. At the diagnosis of clinical T1D, children with a prior early-stage diagnosis had lower median HbA1c (6.8% vs 10.5%), lower median fasting glucose (5.3 mmol/L vs 7.2 mmol/L), and higher median fasting C-peptide (0.21 nmol/L vs 0.10 nmol/L) compared to children without previous early-stage diagnosis. Only 2.5% of those with early-stage diagnosis presented with DKA at diagnosis of clinical T1D [61].

### Prevention of DKA

Rates of DKA at the onset of clinical T1D vary globally, ranging from 15–80%. The INNODIA study reported an overall DKA prevalence of 37% at diagnosis, with rates differing by age: 32.8% in children aged 1–9 years, 43.9% in adolescents aged 10–17 years, and 23% in adults aged 18–45 years [62]. Children who participated in screening programs exhibited lower rates of DKA, HbA1c, fasting blood glucose, and ketonuria at stage 3 T1D diagnosis compared to those without early-stage identification. Additionally, they maintained higher fasting C-peptide levels and required less intensive insulin therapy at diagnosis [61]. Perhaps most importantly, presence of DKA at time of clinical T1D is associated with poorer long-term glycemic control and increases the risk of long-term complications. With early identification and monitoring, the rate of DKA decreases significantly and therefore there is lifelong benefit provided to patients.

### Early Education

Increasing knowledge and awareness of the clinical signs of T1D among patients and families can significantly improve outcomes at diagnosis and long-term disease management. Early education can help to alleviate anxiety, foster healthy habits, and empower individuals with essential knowledge before initiating insulin therapy.

One notable example is The Early Start Study (TESS), an intervention designed to evaluate optimal education and monitoring strategies for children and youth (ages 2–20) with presymptomatic T1D. TESS incorporates continuous glucose monitors (CGMs) and a staged education approach led by diabetes specialists. Families learn to identify blood sugar changes preceding clinical T1D to recognize appropriate timing for insulin initiation. The study aims to maintain blood glucose levels within the target range while reducing the risk of life-threatening DKA at diagnosis [63].

### Preventing Misdiagnosis

Distinguishing between T1D and T2D can be challenging, especially in children due to the increasing prevalence of childhood obesity and in adults where the presentation of T1D may be slowly progressive and atypical. DKA can also occur in T2D, further complicating the clinical picture upon diagnosis. Misdiagnosis of T1D as T2D is common, particularly in adults, with 40% of individuals developing T1D after the age of 30 initially being treated as T2D [64]. This delay in proper diagnosis and insulin initiation can lead to poor glycemic control and increased risk of complications. Given the high prevalence of prediabetes in the general population, it is essential to identify individuals with early stage T1D to ensure timely and appropriate intervention.

### Challenges

Implementing generalized population screening for T1D faces several challenges such as identifying the most effective venues and strategies to integrate screening into routine healthcare. Primary care is a logical setting for screening, but time constraints during visits, already packed with multiple health concerns, often limit the ability to thoroughly explain testing and results. Incorporating IA screening into well-child visits or routine vaccination appointments could streamline the process and fit more seamlessly into existing workflows. Additionally, integrating screening protocols into electronic medical records (EMRs) may enhance efficiency by automating reminders and centralizing results.

Another challenge involves balancing generalized screening efforts with targeted screening. While screening relatives of individuals with T1D or patients with other autoimmune conditions can help identify high-risk individuals, generalized population screening will likely detect more people with autoantibodies who may never progress to clinical T1D, particularly single autoantibody positive individuals. For those identified at stage 1 or stage 2 T1D, the psychological component of knowing they are at risk for progression, combined with the need for ongoing monitoring, can lead to anxiety and challenges with coping. This raises the need for further research to identify additional factors, such as genetic or autoantibody features, that predict progression to clinical T1D. These insights could guide the development of personalized care plans for at-risk individuals.

As screening programs expand, addressing concerns such as resource allocation, public education, and potential anxiety from false positives will also be critical. Streamlining workflows and emphasizing education for health care providers and families can help address some of these barriers and optimize the impact of generalized screening initiatives.

### Monitoring and Follow-Up

While autoantibody screening is critical for early detection of T1D, its benefits depend on consistent follow-up and monitoring. Time from IA positivity to clinical T1D is highly variable, necessitating personalized monitoring approaches. Recently published consensus guidelines recommended monitoring strategies for individuals with pre-stage 3 T1D, which includes periodic measurement of HbA1c, repeated autoantibody testing, self-monitored blood glucose (SMBG), OGTT, and the use of CGM [65]. OGTT is considered the gold standard, however individuals may not be agreeable to repeat OGTT testing. The TEDDY and TrialNet studies demonstrated that a relative increase in HbA1c by ≥ 10% from baseline was just as informative as OGTT for monitoring progression to clinical T1D [66]. CGM data from the ASK study found that children with elevated CGM glucose readings > 140 mg/dL for over 10% of time over a 2-week period have an 80% risk of progressing to clinical T1D within 1 year [67]. Similarly, the DAISY study showed that autoantibody-positive children with 18% or greater CGM time spent at > 140 mg/dL predicts progression to clinical T1D with 100% specificity, 100% PPV, and 75% sensitivity [68].

Close monitoring not only helps to identify progression but also offers opportunities to prevent DKA, access emerging therapies like teplizumab, and participate in interventional trials. However, effective monitoring requires coordination among primary care providers, pediatricians, and endocrinologists along with family members and patients playing an active role in recognizing symptoms and adhering to follow-up. In adults, monitoring is further complicated by slower disease progression and limited research, underscoring the need for studies tailored to this population.

### Cost-Effectiveness of Generalized Autoantibody Testing

The cost-effectiveness of generalized screening programs for T1D is a growing area of interest, driven by the potential to reduce complications and improve long-term health outcomes. Key factors influencing the feasibility of generalized screening include the prevalence of T1D, test costs (which vary across regions), and economic benefits from reducing DKA rates and long-term complications, and the potential benefit of immunomodulatory therapy through earlier intervention.

The ASK and Fr1da studies have provided valuable insights into screening costs and implementation. In the ASK study, the cost per T1D case detected was $4,700, much lower than the $14,000 associated with routine clinical diagnosis. Cost-effectiveness in this model depended on reducing lab costs to at least $80 per person tested along with a 20% reduction in DKA events at diagnosis and 0.1% lifetime improvement in HbA1c [69]. The Fr1da study reported costs of €28.17 ($32 USD) per child screened and €9,117 ($10,345 USD) per presymptomatic T1D case detected. While the per-case cost in Fr1da appears higher than in ASK, these figures are not directly comparable. The ASK study was based on modeled projections assuming system-level efficiencies, while Fr1da reflects real-world costs within a public health infrastructure. Additionally, the Fr1da study negotiated a cost of €1.20 ($1.36 USD) per sample using the 3-Screen Islet Cell Antibody ELISA, highlighting the importance of cost-efficient lab testing in making generalized screening viable across healthcare systems [70]. These findings suggest that presymptomatic screening could be particularly effective in areas with high DKA prevalence and well-established screening infrastructures, if long-term benefits and improved HbA1c levels are sustained over time.

Population-based screening programs continue to be developed, with some favoring targeted approaches (e.g., family history or through genetic risk) compared to be universal screening. Alternative emerging strategies include combining T1D screening with tests for other autoimmune conditions, such as celiac disease, to optimize resource utilization. To enhance the cost-effectiveness of generalized screening programs, ongoing research focuses on improving risk stratification methods, integrating genetic screening, and negotiating lower laboratory costs. Such advances could make early detection more accessible while maximizing the clinical and economic benefits of these programs. Additionally, the advent of disease modifying therapies, such as teplizumab, may shift cost-effectiveness models by extending the time to insulin dependence and reducing complications.

## Potential Shifts in Policy for Screening and Future Directions

The American Diabetes Association (ADA) currently recommends T1D screening for individuals with a family history of the disease and for those without a family history who participate in research studies, including children with high GRS. Efforts are now expanding towards generalized population screening to capture the ~ 90% of individuals with T1D who lack a family history. Implementing such programs effectively requires collaboration among primary care providers, endocrinologists, diabetes educators, and public health experts. However, primary care providers will need structured training and resources to discuss screening, while endocrinologists may need to engage at earlier disease stages, depending on patient and provider comfort levels.

Community engagement and education are pivotal in encouraging participation in screening programs. Public awareness campaigns, concise education materials, and social media outreach could emphasize the importance of early detection. STOP T1D, led by the Barbara Davis Center for Diabetes, has resources for education and awareness. Meanwhile, organizations like BreakthroughT1D are advocating for policy changes, such as obtaining a United States Preventive Services Task Force (USPSTF) recommendation for T1D screening. A positive recommendation could ensure insurance coverage for screening without out-of-pocket costs. Additionally, collaboration with other organizations such as the ADA, Endocrine Society, American College of Physicians (ACP), American Association of Pediatrics (AAP),and Bright Futures could support provider education and public outreach initiatives. Patient advisory committees within screening programs are crucial for providing feedback to refine implementation strategies.

## Conclusion

Advancements in early screening and detection of T1D through autoantibody detection offer a transformative opportunity to revolutionize disease management by identify at-risk individuals before clinical onset. Screening programs have demonstrated significant benefits, including earlier diagnosis, reduced rates of DKA, and improved long-term glycemic control. These efforts represent a promising shift from reactive treatment to proactive prevention, fostering better health outcomes and empowering patients and families through early education and intervention.

While these developments are encouraging, further research is needed to refine and expand the reach of what has been established. Notably, there is a small but important subset of patients who progress to stage 3 T1D without ever developing autoantibodies. Further characterization of the role and the affinity of each autoantibody will also continue to contribute to risk prediction but may need to be aided by additional measures beyond dysglycemia to better define the timing of immune activity against beta cells. Finally, to successfully roll out generalized population screening, key areas of focus include standardizing autoantibody assays for widespread use, ensuring cost-effectiveness, and addressing health disparities that limit equitable access. Current study cohorts often lack racial and ethnic diversity, underscoring the need for further research that incorporates diverse populations to enhance generalizability. Additionally, questions remain about the natural history of T1D in both children and adults, including whether autoantibodies develop later in life with adult-onset cases or are present in childhood with prolonged latency. Expanding research into adult screening protocols alongside pediatric programs can ensure a more comprehensive approach to early detection.

## Key References

- Phillip M, Achenbach P, Addala A, et al. Consensus Guidance for Monitoring Individuals With Islet Autoantibody-Positive Pre-Stage 3 Type 1 Diabetes. Diabetes Care. 2024;47(8):1276–1298. https://doi.org/10.2337/dci24-0042.
  - This consensus guideline provides recommendations for monitoring individuals with IA + pre-stage 3 T1D, emphasizing standardized surveillance strategies to optimize early detection and timely intervention. Broad advice includes partnerships between endocrinologists and primary care providers, confirmation of autoantibody positivity with a second sample, periodic medical monitoring of IA + individuals (glucose levels, education on symptoms, psychosocial support). Interested people should be offered trial participation or approved therapies.
- Felton JL, Redondo MJ, Oram RA, et al. Islet autoantibodies as precision diagnostic tools to characterize heterogeneity in type 1 diabetes: a systematic review. Communications medicine. 2024;4(1):66. https://doi.org/10.1038/s43856-024–00478-y.
  - This systematic review highlights the role of islet autoantibodies in diagnosis, illustrating how different IA profiles contribute to understanding T1D heterogeneity and disease progression. Findings support continued use of pre-clinical staging paradigms based on IA number and suggest that additional IA features, such as age and genetic risk, could offer more precise stratification.
- Sims EK, Besser REJ, Dayan C, et al. Screening for Type 1 Diabetes in the General Population: A Status Report and Perspective. Diabetes. 2022;71(4):610–623. https://doi.org/10.2337/dbi20-0054.
  - This report outlines the current status of general population screening for T1D, discussing the potential benefits, challenges, and future directions for large-scale screening implementation. It reviews the background of the efforts from the March 2021 T1D TrialNet Screening Committee meeting, a session held to discuss ongoing efforts in general population screening.
- Ghalwash M, Anand V, Ng K, et al. Data-Driven Phenotyping of Presymptomatic Type 1 Diabetes Using Longitudinal Autoantibody Profiles. Diabetes Care. 2024;47(8):1424–1431. https://doi.org/10.2337/dc24-0198.
  - This study uses data-driven phenotyping of longitudinal autoantibody profiles to identify distinct patterns of T1D progression, offering insights into personalized disease risk assessment. They identified five main clusters of distinct autoantibody profiles and progression rates to T1D.

## Data Availability

No datasets were generated or analysed during the current study.

## Declarations

Z.Q. has acted as a scientific advisor to Sanofi for a scientific advisory board in November 2023.
